# Prevalence and Risk Factors of Inappropriate Drug Dosing among Older Adults with Dementia or Cognitive Impairment and Renal Impairment: A Systematic Review

**DOI:** 10.3390/jcm13195658

**Published:** 2024-09-24

**Authors:** Saad Alhumaid, Woldesellassie M. Bezabhe, Mackenzie Williams, Gregory M. Peterson

**Affiliations:** School of Pharmacy and Pharmacology, University of Tasmania, Hobart 7000, Australia; woldesellassie.bezabhe@utas.edu.au (W.M.B.); mackenzie.williams@utas.edu.au (M.W.); g.peterson@utas.edu.au (G.M.P.)

**Keywords:** cognitive impairment, dementia, kidney disease, inappropriate drug dosing, older adults, prevalence, renal impairment, systematic review

## Abstract

**Background:** Potentially inappropriate medication prescribing is prevalent and well studied in older adults. However, limited data are available on inappropriate drug dosing in those with dementia or cognitive impairment and renal impairment. **Objectives:** We aimed to examine the prevalence of, and factors associated with, inappropriate drug dosing in older patients with dementia or cognitive impairment and renal impairment. **Methods:** We adhered to the Preferred Reporting Items for Systematic Reviews and Meta-Analyses (PRISMA) guideline and the Cochrane Handbook for Systematic Reviews of Interventions. We searched Medline, Embase, CINAHL, and PubMed for studies on inappropriate drug dosing in older patients with dementia or cognitive impairment and renal impairment, published from 1 January 2000 to 31 August 2024, with English language restriction following the PICOS search strategy. Two reviewers independently screened all titles and abstracts, extracted data from included studies, and undertook quality assessment using the Joanna Briggs Institute (JBI) tool. Descriptive statistics were used to summarise and present findings. **Results:** In total, eight retrospective cohort studies were included. Of the total number of patients with dementia who had renal impairment (*n* = 5250), there were 2695 patients (51.3%; range: 0–60%) who had inappropriate drug dosing. Drugs commonly prescribed in inappropriate doses in patients with dementia who had renal impairment included memantine, baclofen, nonsteroidal anti-inflammatory drugs (NSAIDs), metformin, digoxin, morphine, and allopurinol. The studies did not identify statistically significant risk factors for inappropriate drug dosing. **Conclusions:** Inappropriate drug dosing among older adults with dementia or cognitive impairment and renal impairment appears to occur frequently. While our findings should be interpreted with caution owing to the small number of studies and substantial heterogeneity, proactive prevention, recognition, and management of inappropriate drug dosing in this population is warranted.

## 1. Introduction

Dementia or cognitive impairment and renal impairment become more common with advanced age [1]. The prevalence of multiple diseases, polypharmacy, and age-related physiological changes affecting pharmacokinetics and pharmacodynamics makes drug therapy in older individuals complicated [2]. One of the challenges of drug therapy in old age is the functional alterations in the central nervous system and increased number of medications used to treat dementia, inevitably increasing the likelihood of drug-related problems (including adverse drug reactions, adverse drug events, and medication errors) [3]. In a recent systematic review that included 15 studies, medication misadventure and potentially inappropriate medication prescribing in older adults with dementia or cognitive impairment were found to be common [4]. The prevalence of drug-related problems ranged from 9.1% to 83.6% and the prevalence of the use of potentially inappropriate medications ranged from 23.7% to 73.5% [4].

A common cause of adverse events in patients is failure to properly adjust doses for renal insufficiency, and the risk of these adverse events is unsurprisingly higher in older adults [5,6]. For instance, older adults with pre-existing renal impairment may be particularly susceptible to nephrotoxicity; therefore, renal function monitoring should be considered in patients using a medicine that may impair renal function or cause nephrotoxicity [7]. One systematic review found the prevalence of inappropriate prescribing among adults with chronic kidney disease (CKD) ranged between 9.4% and 81.1% and 13% and 80.5% in hospital and ambulatory settings, respectively [8]. Another systematic review that investigated drug-related problems among hospitalised patients with CKD found that the prevalence of these problems ranged between 12% and 87% [9].

To date, some systematic reviews have been conducted to evaluate the inappropriate prescribing and drug dose adjustment among older adults with either dementia or renal impairment [8,10,11,12,13,14]. The findings varied widely and individual studies examined different classes of medications [8,10,11,12,13,14]. Moreover, none of the reviews performed sub-group analysis for patients with both dementia and CKD. Due to the lack of comprehensive and updated systematic reviews focusing on potentially inappropriate drug dosing in older adults with dementia or cognitive impairment and renal impairment, we aimed to estimate the prevalence of inappropriate drug dosing, and examine the medications and factors associated with inappropriate drug dosing, in older patients with dementia or cognitive impairment and renal impairment.

## 2. Methods

### 2.1. Design

This systematic review was conducted based on the Preferred Reporting Items for Systematic Reviews and Meta-Analyses (PRISMA) statement [15] and the Cochrane Handbook for Systematic Reviews of Interventions [16]. The systematic review protocol was entered into the International Prospective Register of Systematic Reviews (PROSPERO) under CRD42023454239.

The review was planned considering PICOS criteria, which provide a structured framework in systematic reviews to define the research question and guide the search strategy by outlining the Population, Intervention, Comparison, Outcome, and Study Design. This approach ensures a comprehensive and focused search, enabling the identification and selection of relevant studies for inclusion in the review [17]. The PICOS table can be seen in Table 1.

The PRISMA 2020 checklist was used (see Supplementary Materials) [15].

### 2.2. Data Sources and Search Strategy

Four electronic databases (Medline, Embase, CINAHL, and PubMed) were searched from 1 January 2000 to 31 August 2024 in the English language. The following keywords, alone or in combination, were used: *dosing* OR *dosage* OR *dose* AND *kidney impairment* OR *renal impairment* OR *kidney insufficiency* OR *renal insufficiency* OR *kidney disease* OR *renal disease* OR *kidney function* OR *renal function* OR *renal problem* OR *kidney problem* OR *glomerular filtration rate* OR *GFR* PLUS *dementia* OR *Alzheimer disease* OR *cognitive impairment* OR *aphasia* OR *Lewy body disease*. The literature search was carried out using natural language keywords and, where applicable, medical subject headings (MeSH) terms (see Supplementary Tables S1 and S2 for database-specific search strategies and database-specific search syntax). Results from each database search were saved individually using EndNote 21.2 (Clarivate, Philadelphia, PA, USA, 2023) prior to being exported to Covidence and combined in one library for screening by three independent researchers, after removing duplicates. Articles reporting inappropriate drug dosing, based on renal function, among older adults with dementia or cognitive impairment and renal impairment were initially selected using the title and abstract. While our literature review focused predominantly on inappropriate drug dosing among older patients with dementia, some studies combined dementia and cognitive impairment, so our review included older adults with dementia or cognitive impairment.

### 2.3. Inclusion/Exclusion Criteria

We included observational studies that reported real-world inappropriate drug dosing of any medications in older adults (≥65 years old) with dementia or cognitive impairment and renal impairment. We excluded editorials, conference abstracts, theses, commentaries, letters to the editor, drug trials, case reports, case series (if including less than 5 patients), qualitative studies, expert opinions, discussion papers, reviews or meta-analyses, or studies available in languages other than English.

### 2.4. Definitions of Renal Impairment and Inappropriate Drug Dosing

Renal impairment was defined as having an estimated glomerular filtration rate (eGFR) <60 mL/min/1.73 m^2^ [18]. Studies that we included must have determined the eGFR of patients via creatinine-based equations, including the Cockcroft-Gault (CG) equation [19], the Modification of Diet in Renal Disease (MDRD) [20], or the Chronic Kidney Disease Epidemiology Collaboration (CKD-EPI) [21].

Inappropriate drug dosing among older patients with dementia or cognitive impairment and renal impairment was defined as the use of prescribed medications at the incorrect dosage and/or prescribing of medications that are contraindicated based on the patient’s renal function. This was evaluated based on noncompliance with guidelines or product information, as specified by each study. Prescriptions not requiring dosage adjustment were classified as having appropriate doses relative to renal function [22,23,24].

### 2.5. Data Extraction

Two reviewers independently assessed the studies and excluded those that did not meet the inclusion criteria based on their title and/or abstract. If there was disagreement and agreement could not be reached after discussion between the two investigators, a third investigator adjudicated. The full text of each potentially eligible study was retrieved by one member of the research team. A full-text review of publications was independently conducted by two research team members (from SA, GP, WB) and those meeting the inclusion criteria were retained for data extraction and analysis. Any disagreements over the eligibility of particular studies were resolved through discussion with a third reviewer. The reasons for a paper being excluded were recorded.

A standardised data collection form was used to collate information and facilitate study quality assessment and data analysis. The following data were extracted from selected studies: authors, publication year, study location, study design, healthcare setting and source of data, methods and resources used to detect inappropriate drug dosing, study size, number of patients with dementia or cognitive impairment, medications examined, number of patients with dementia or cognitive impairment who had renal impairment, number and the percentage of patients with dementia or cognitive impairment who had renal impairment and inappropriate drug dosing, and number and the proportion of patients with dementia or cognitive impairment who had renal impairment and inappropriate drug dosing among different medications or classes of medications.

### 2.6. Quality Assessment

The Joanna Briggs Institute (JBI) checklist for cross-sectional studies was used to assess the quality of the studies with the following scoring criteria: ≥6 scores = high quality, 4–5 scores  =  moderate quality, and  ≤3 scores  =  low quality [25]. Retained papers were independently assessed for quality by two research team members (from SA, WB, GP). A third reviewer (MW) acted as adjudicator if an agreement could not be reached by discussion (see Supplementary Table S3 for quality assessment of included studies).

### 2.7. Data Analysis

We primarily examined the prevalence of inappropriate drug dosing, associated medications, and factors associated with inappropriate drug dosing in older patients with dementia or cognitive impairment and renal impairment. Descriptive statistics were used to describe the data. For categorical variables, frequencies and percentages were used to summarise the data. Microsoft Excel 2019 (Microsoft Corp., Redmond, DC, USA) was used for all statistical analyses.

## 3. Results

**Figure 1:**
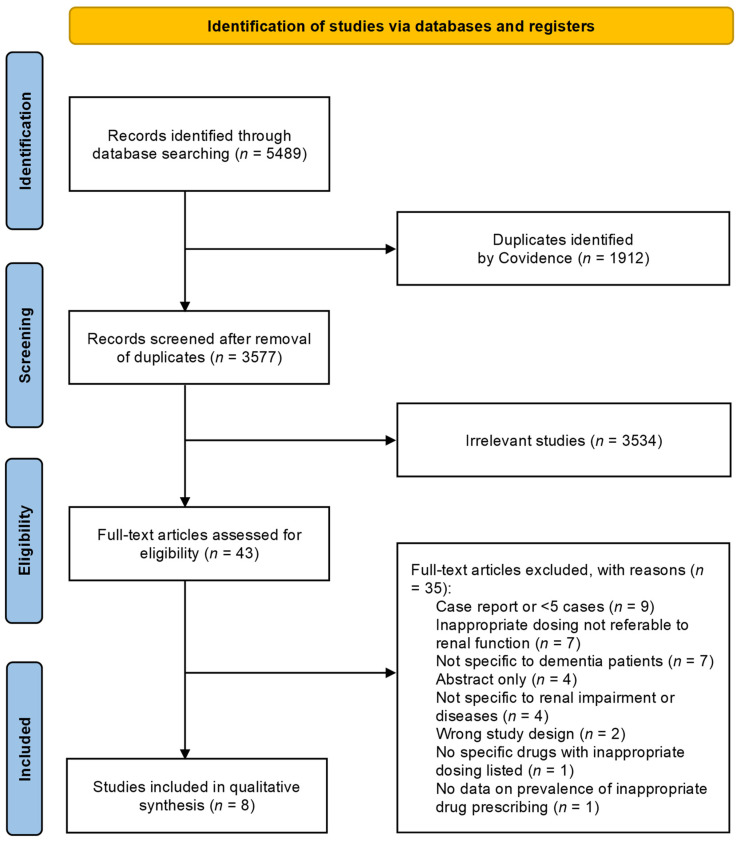
Flow diagram of studies included in the systematic review.

Our initial search uncovered 5489 articles potentially relevant to the research question. After eliminating duplicates, we assessed the eligibility of 3577 articles based on their abstracts. From these, 3534 irrelevant studies were excluded. Following a meticulous full-text review of the remaining 43 articles, an additional 35 were excluded. The reasons for excluding the remaining 35 articles are detailed in the Supplementary Table S4. This screening process yielded a final selection of eight retrospective cohort studies for inclusion in this systematic review [22,23,24,26,27,28,29,30]. The results of the literature search are shown in Figure 1.

The detailed characteristics of the studies that we included are shown in Table 2. The studies were conducted in the United States (*n* = 3) [22,27,30], Sweden (*n* = 3) [23,24,29], the United Kingdom (*n* = 1) [26], and Canada (*n* = 1) [28]. All were multi-centre studies, except for one single-centre study [27]. Two studies were deemed to have high methodological quality (JBI scores ≥6) [26,29], five studies had moderate methodological quality (JBI scores between 4 and 5) [22,23,24,28,30], and the remaining study had low methodological quality (JBI score ≤3) [27]. Four studies were based solely on data from nursing home residents [22], general practice [26,29], or inpatient medical records in hospitals [27]. Three studies were based on hospitals and nursing homes [23,24] or general practice and hospitals [30]. Only one study was based on a nationwide administrative database [22]. One study reported patients who resided in rural or metropolitan residential areas (*n* = 1728) [26] and one study reported patients who resided in metropolitan residential areas (*n* = 9) [27], while six studies did not report the patients’ region of residence [22,23,24,28,29,30]. Three studies included patients who participated in previous research projects: the Stockholm CREAtinine Measurements (SCREAM) study [29,31], the Atherosclerosis Risk in Communities (ARIC) study [30,32], and a randomised controlled trial investigating pharmacist participation in hospital ward teams and hospital readmission rates among people with dementia [23,33].

Two studies were based on older people with either dementia or cognitive impairment [23,24] and six solely on patients with dementia [22,26,27,28,29,30]. Five studies involved patients diagnosed based solely on these screening tests: Mini-Mental State Examination (MMSE) [27,30], International Classification of Diseases, 10th Revision (ICD-10) [26,29], and International Classification of Diseases, 9th Revision (ICD-9) [22]. Other studies involved patients with dementia or cognitive impairment based on MMSE and information related to memory, orientation, or executive function [23,24] or ICD-10, Oral Health Impact Profile (OHIP), and Diagnostic and Statistical Manual of Mental Disorders, 4th edition (DSM-IV) [28]. Four studies looked at patients with the following specific types of dementia: Alzheimer’s disease (*n* = 4) [23,24,27,30], vascular dementia (*n* = 2) [23,24], and Lewy body dementia (*n* = 1) [23].

Each study included patients with all CKD stages [22,23,24,26,27,28,30], except for one study that was conducted on patients with either CKD stage 3 or CKD stage 4 [29]. Renal function was assessed using eGFR (*n* = 5) [22,26,28,29,30] or creatinine clearance (CrCl) (*n* = 3) [22,23,24]. Three studies solely used the CKD-EPI Collaboration equation [28,29,30], and two studies solely used the CG equation [23,24], to calculate the renal function. One study utilised two equations: the MDRD and CG equations [22].

Inappropriate drug dosing was detected by using literature evidence from systematic reviews (baclofen and morphine only) [24,28,34]; STOPP Version 2 criteria [26,35]; a summary of product characteristics (memantine only) [27,36]; consensus guidelines for oral dosing of primarily renally cleared medications in older adults [22,37]; US Department of Veterans Affairs/Department of Defence CKD guidelines [22,38]; classification of drug-related problems by the modified version of criteria outlined in Cipolle et al. [23,39]; Swedish criteria and inappropriate drug classification according to renal function and disease [23,40]; the Janusmed Drugs and Renal function system [29,41]; Micromedex [30,42]; and Geriatric Dosage Handbook guidelines [24,43].

Only two of the studies, which had small samples with dementia or cognitive impairment, did not make any restrictions on the drugs examined in their research [23,24], while two studies only looked at baclofen [28] or memantine [27]. The other studies looked at restricted lists of drugs [22,26,29,30]. The drugs most commonly examined were anti-dementia drugs (memantine and galantamine) [22,23,24,27,29,30], nonsteroidal anti-inflammatory drugs (NSAIDs) [23,24,26], anti-diabetic drugs (metformin and glibenclamide) [23,24,26], diuretics and anti-hypertensive drugs (bendroflumethiazide, enalapril, hydrochlorothiazide, and spironolactone) [23,24], antibiotics (amoxicillin, ampicillin, cefotaxime, ciprofloxacin, and nitrofurantoin) [23,24], opioids (morphine and tramadol) [23,24], digoxin [23,26], and allopurinol [23,24] (see Supplementary Table S5 for a complete list of medications examined, including dosage recommendations for their appropriate prescribing according to renal function) [44,45,46,47,48,49,50,51,52,53,54,55,56,57,58,59,60,61,62,63,64,65].

Of the total number of patients with dementia who had renal impairment (*n* = 5250), there were 2695 patients (51.3%; range 0–60%) who had inappropriate drug dosing (26,28–30]; however, this estimate was predominantly influenced by two large studies [(26,28–30], one of which examined only baclofen. Drugs commonly prescribed in inappropriate doses in patients with dementia or cognitive impairment who had renal impairment included memantine, baclofen, nonsteroidal anti-inflammatory drugs, metformin, digoxin, morphine, and allopurinol. The proportions of drugs implicated in inappropriate dosing according to renal function are provided in Table 2. Two studies with evidence on inappropriate drug dosing for single medications, namely baclofen and memantine, were among those that reported the highest prevalence of inappropriate dosing (51.6% and 54.2%, respectively) [28,29]. On the other hand, three studies related to single therapeutic agents did not show a high prevalence of inappropriate drug dosing: (memantine: 1.1% and 12.8%) [17,22] and (galantamine: 0) [25].

Although three studies reported on the incidence of adverse health outcomes associated with potentially inappropriate prescribing [23,26,28], only one study reported symptoms or diagnoses induced specifically by the inappropriate drug dosing in relation to renal function [28]. Inappropriate dosing of baclofen ( ≥20 mg/day) in patients with CKD was associated with a higher 30-day risk of hospitalisation with encephalopathy (defined as delirium, disorientation, transient ischemic attack and related syndromes, alteration of awareness, or unclear diagnosis of dementia), but not all-cause mortality [28]. The final treatment outcome was reported in four studies, and all the patients with dementia or cognitive impairment who received inappropriate drug dosing survived [23,24,28,29].

Only one study specifically reported the risk factors for inappropriate drug dosing among older adults with renal impairment and dementia or cognitive impairment [24]. None of the factors examined proved to be statistically significant, although two were close to being significant: living in nursing homes [adjusted odds ratio (aOR): 1.88 (95% confidence interval (CI) 0.97–3.66)] and increasing age [aOR: 1.04 (95% CI 0.99–1.10)].

## 4. Discussion

To our knowledge, this is the first systematic review to examine the prevalence of inappropriate drug dosing in older people with dementia or cognitive impairment and renal impairment. In this review, we included 5250 patients aged ≥65 years old with dementia and renal impairment from eight observational studies. This review shows that there is still a paucity of studies in this crucial field of research. The overall estimated prevalence of potentially inappropriate drug dosing among older adults with dementia and renal impairment from nursing homes, general practice, or hospitals was high (51.3%); however, we acknowledge that this estimate was predominantly influenced by two large studies [26,28,29,30], one of which examined only baclofen.

The lack of systematic reviews to report the prevalence of inappropriate drug dosing in older adults with dementia or cognitive impairment and renal impairment makes it reasonable to compare our review findings with publications that involved older adult patients with renal impairment without dementia or cognitive impairment. Our pooled prevalence of inappropriate drug dosing in older people with dementia and renal impairment was higher than that of a systematic review reporting a prevalence between 16% and 37.9% for inappropriate prescribing in adults with CKD living in long-term care facilities [8]. This variation may relate to differences in the assessment of inappropriate drug dosing, study design, setting, and population characteristics. However, the prevalence of inappropriate drug dosing in older people with dementia or cognitive impairment and renal impairment may also be underreported, as this population generally has a reduced ability to communicate symptoms or diagnoses explicitly induced by the inappropriate drug dosing [66,67].

A precise prevalence figure for potentially inappropriate drug dosing in older adults with either dementia or cognitive impairment and renal impairment was not possible to determine because of methodological and reporting differences across studies. One of the main reasons was the different groups of drugs examined between studies. There was also the use of different equations to estimate the renal function. Three studies included in this review used the proportion of inappropriate drug dosing calculated by the CKD-EPI equation [28,29,30]. Other equations such as the CG and MDRD were utilised to calculate GFR or CrCl [22,23,24]. For example, in the study by Hanlon et al., the median estimated creatinine clearance via the CG equation was 67 mL/min/1.73 m^2^, whereas it was 80 mL/min/1.73 m^2^ with the MDRD [22]. As a result, the prevalence of potentially inappropriate prescribing of at least one renally cleared medication was 11.9% and 6.0% according to the CG and MDRD equations, respectively [22].

Differences in the reported prevalence of inappropriate drug dosing may also be explained by the use of different methods by prescribers and authors to detect inappropriate dosing. For example, we found variable prevalences of inappropriate drug dosing for metformin in older adults with dementia or cognitive impairment and renal impairment, as recommendations on dosage limits for metformin differed between tools and prescribing guidelines (24%, 19.7%, and 12.5%) [23,24,26]. Delgado’s team calculated the prevalence of potentially inappropriate drug dosing for metformin to be 19.7% using STOPP Version 2 criteria [26], but when using the Swedish criteria and inappropriate drug classification according to renal function, the prevalence increased to 24% [23]. The higher prevalence of inappropriate dosing for metformin found by Sönnerstam and his colleagues compared to the rate reported by Delgado et al. can be explained, in part, by the use of Geriatric Dosage Handbook guidelines that contraindicate the use of metformin for patients with a GFR <60 mL/min [24,43]. Despite the contraindication for metformin use in diabetic patients with severe renal impairment [68,69], some guidelines still recommend low doses of metformin in patients with GFRs <30 mL/min, which would change the prevalence of inappropriate drug dosing of this commonly prescribed medication [70]. In the absence of any evidence for an increased risk of lactic acidosis in patients with a GFR <30 mL/min [71,72], some physicians may choose to use metformin in selected people with GFRs <30 mL/min where they perceive that the benefits outweigh the risks [70].

Of all the drugs studied, those found to be most commonly prescribed in inappropriate doses in patients with dementia who had renal impairment included NSAIDs, memantine, and baclofen. NSAIDs are one of the most commonly used medications in older adults with dementia or cognitive impairment [73]. Apart from the risk of gastrointestinal, renal, and cardiovascular adverse events, reported side effects of NSAIDs in older people include cognitive dysfunction, particularly with the use of indomethacin [74]. Such side effects are concerning in any older adult population, but especially among those with dementia or cognitive impairment, and the safe use of NSAIDs should depend upon a thorough patient evaluation for characteristics that increase the risk of developing NSAID-induced toxicity [75].

A recent systematic review that sought to identify medication misadventure among patients with dementia or cognitive impairments reported memantine as being commonly linked to drug-related problems [4]. Abnormally high plasma concentrations of memantine were reported to place patients at an increased risk for side effects, including confusion, agitation, insomnia, dizziness, and headaches [76]. Prescribers and pharmacists should pay special attention to the dose of memantine in older, frail patients with compromised renal function.

In a large study that focused solely on baclofen use, Muanda et al. reported that 945 patients (51.6%) with dementia and CKD were prescribed inappropriate doses of baclofen based on renal function [28]. Patients with CKD can develop toxicity within 3 days of baclofen initiation [48,77,78]. Baclofen toxicity causes a wide range of manifestations that appear clinically as somnolence, myoclonus, hypotonia, bradycardia, hypotension, respiratory depression or failure, seizures and/or coma [79]. Muanda et al. showed an association between inappropriate drug dosing of baclofen and an increased risk of hospital admission with encephalopathy, but not all-cause mortality, in patients with dementia and reduced renal function [28]. Baclofen has been newly added as a drug that should be avoided or have its dosage reduced with decreased kidney function (eGFR <60 mL/min/1.73 m^2^) in the American Geriatrics Society 2023 updated AGS Beers Criteria^®^, based on the risk of encephalopathy [28,80,81,82].

Most clinical outcomes associated with inappropriate drug dosing are potentially preventable, and implementation of strategies to minimise the preventable adverse outcomes associated with inappropriate drug dosing in this vulnerable group of patients is warranted [83,84]. Deprescribing, regular review of medications and renal function, and better communication between healthcare providers are some examples of such strategies. These strategies should target the medicines commonly prescribed to older adults with dementia or cognitive impairment and renal impairment [85,86,87]. Healthcare organisations should pay attention to this problem in the older population with dementia and renal impairment, given the well-established effect that inappropriate use of primarily renally cleared drugs has on morbidity, mortality, and healthcare costs [30,88,89].

### Strengths and Limitations

To the best of our knowledge, this is the first systematic review conducted to review the available literature on inappropriate drug dosing in older patients with dementia or cognitive impairment and renal impairment. Screening, data extraction, and quality assessment were performed in accordance with the PRISMA guideline and the Cochrane Handbook for Systematic Reviews of Interventions.

We acknowledge that our review was not without some limitations. Firstly, we only included eight publications from four countries. Also, some of the studies had small sample sizes. The generalisability of the results could therefore be limited. Secondly, direct comparison between studies was limited and meta-analysis was not performed due to clinical and methodological heterogeneity, including the methods used to assess renal function for drug dosing purposes. Thirdly, we only focused on the studies that were in the English language. Fourthly, all studies included in this review were retrospective in design, which could have introduced potential reporting bias due to reliance on data from clinical records.

## 5. Conclusions

Inappropriate drug dosing among older adults with dementia or cognitive impairment and renal impairment appears to occur frequently. Proactive recognition and management of inappropriate drug dosing in this population is advisable, and drugs warranting close attention include NSAIDs, memantine, and baclofen. The results should be interpreted with some caution, owing to the small number of studies and substantial heterogeneity, and indicate a need for future research in this area.

## Figures and Tables

**Table 1:** PICOS table for the search strategy in prevalence and risk factors of inappropriate drug dosing among older adults with dementia or cognitive impairment and renal impairment.

Component	Description
Population (P)	Studies related to dementia or cognitive impairment with co-existing renal impairment in older patients (≥65 years old).
Intervention (I)	Not applicable.
Comparison (C)	Not applicable.
Outcomes (O)	Identification of the prevalence of inappropriate drug dosing, and examination of the medications and factors associated with inappropriate drug dosing.
Study Design (S)	The review encompasses a variety of study designs, such as cohort studies and cross-sectional studies, to ensure a comprehensive synthesis of relevant evidence.

**Table 2:** Summary of the characteristics of the included studies with evidence on inappropriate drug dosing among older adults with dementia or cognitive impairment and renal impairment.

Author, Year, Study Location	Study Design	Healthcare Settings	Source of Data	Methods and Resources Used to Detect Inappropriate Drug Dosing	Study Size (n)	Dementia/Cognitive Impairment Reported (%)	Medications Examined	Patients with Dementia Who Had Renal Impairment, n	Patients with Dementia or Cognitive Impairment Who Had Renal Impairment and Inappropriate Drug Dosing, n (%)	Proportion of Patients with Dementia or Cognitive Impairment Who Had Inappropriate Drug Dosing Among Different Medications or Classes of Medications According to Renal Function (%)	Assessment of Study Quality
Delgado et al. 2021 [26], United Kingdom	Retrospective cohort, multi-centre	Community, nursing homes or aged care facilities	Primary care records via CPRD, UK	STOPP Version 2 criteria [34]	54,638	11,175 (20.4)	NSAIDs Metformin Digoxin Direct thrombin inhibitors (e.g., dabigatran) Colchicine Factor Xa inhibitors (e.g., rivaroxaban, apixaban)	3292	1728 (52.5) (dementia only)	NSAIDs: 1322/1728 (76.5) * Metformin: 340/1728 (19.7) * Digoxin: 43/1728 (2.5) * Direct thrombin inhibitors (e.g., dabigatran): 22/1728 (1.3) * Colchicine: 1/1728 (0.06) *	High
Dolder et al. 2009 [27], United States	Retrospective cohort, single centre	Hospitals	Patients’ medical charts, geriatric psychiatry inpatient ward, Carolina, US	Summary of product characteristics (memantine only) [35]	70	70 (100)	Memantine (*n* = 70)	15	9 (60) (dementia only)	Memantine (oral dose at ≥10 mg/day): 9/70 (12.8)	Low
Hanlon et al. 2011 [22], United States	Retrospective cohort, multi-centre	Nursing homes	MDS data, any one of the 133 VAs nursing homes, US	Consensus guidelines for oral dosing of primarily renally cleared medications in older adults [36] and DoVA/DoD CKD guidelines [37]	1304	437 (33.5)	Memantine (*n* = 90)	Not possible to extract	1 (dementia only)	Memantine (oral dose at ≥10 mg/day): 1/90 (1.1) ^¶^	Moderate
Muanda et al. 2019 [28], Canada	Retrospective cohort, multi-centre	General population	Linked administrative health care databases at the ICES, Ontario, Canada	Based on an SR to determine the median dose reported in cases of baclofen toxicity in patients with CKD (baclofen only) [28]	15,942	1830 (11.5)	Baclofen (*n* = 1830)	1830	945 (51.6) (dementia only)	Baclofen (oral dose at ≥20 mg/day): 945/1830 (51.6)	Moderate
Pfister et al. 2017 [23], Sweden	Retrospective cohort, multi-centre	Hospitals and nursing homes	Data for patients admitted to acute internal medicine ward, orthopedic clinic, Umeå University Hospital and medicine wards, County Hospital, Skellefteå, Sweden	Classification of drug-related problems by the modified version of criteria in Cipolle et al. [38] and Swedish criteria and inappropriate drug classification according to renal function and disease [39]	212	212 (100)	No restrictions. OTC drugs were excluded	NR	24 (dementia or cognitive impairment)	Memantine: 3/24 (12.5) * Digoxin: 3/24 (12.5) * Metformin: 3/24 (12.5) * Glibenclamide: 2/24 (8.3) * Morphine: 2/24 (8.3) * Tramadol: 2/24 (8.3) * Allopurinol: 1/24 (4.2) * Enalapril: 1/24 (4.2) * Fondaparinux: 1/24 (4.2) * Glipizide: 1/24 (4.2) * Sucralfate: 1/24 (4.2) * Hydrochlorothiazide: 1/24 (4.2) * Ibuprofen: 1/24 (4.2) * Ketoprofen: 1/24 (4.2) * Nitrofurantoin: 1/24 (4.2) *	Moderate
Schmidt-Mende et al. 2019 [29], Sweden	Retrospective cohort, multi-centre	Community	Primary care records via SCREAM database, a repository of laboratory data of individuals, Stockholm, Sweden	Janusmed Drugs and Renal function version 2016 [40]	32,533	1353 (4.1)	Subset of 50 drugs (CKD stage 3) and subset of 66 drugs (CKD stage 4). Only memantine could be examined in patients with dementia (*n* = 24)	108	13 (12) (dementia only)	Memantine (oral dose at ≥10 mg/day): 13/24 (54.2)	High
Secora et al. 2018 [30], United States	Retrospective cohort, multi-centre	Community and hospitals	Data extracted from the ARIC study, a project based on laboratory and physical examination data, four US communities	Micromedex [41]	6392	5 (0.1)	Subset of 554 drugs. Only galantamine could be examined in patients with dementia (*n* = 5)	5	0 (0)	Galantamine (use if eCrCl <9 mL/min): 0/5 (0)	Moderate
Sönnerstam et al. 2016 [24], Sweden	Retrospective cohort, multi-centre	Hospitals and nursing homes	Data for patients admitted to acute internal medicine or orthopaedic ward, Norrland University Hospital and medical ward, County Hospital, Skelleftea°, Sweden	Geriatric Dosage Handbook guidelines [42] and an SR on the use of opioid medication for those with moderate to severe cancer pain and renal impairment (morphine only) [43]	428	428 (100)	No restrictions	Not possible to extract	50 (dementia or cognitive impairment)	Metformin: 12/50 (24) * Allopurinol: 8/50 (16) * Morphine: 5/50 (10) * Spironolactone: 3/50 (6) * Nitrofurantoin: 3/50 (6) * Hydrochlorothiazide: 2/50 (4) * Glibenclamide: 2/50 (4) * Alendronate: 2/50 (4) * Memantine: 2/50 (4) * Ketoprofen: 1/50 (2) * Tramadol: 1/50 (2) * Raloxifene: 1/50 (2) * Alfuzosin: 1/50 (2) * Amoxicillin: 1/50 (2) * Ampicillin: 1/50 (2) * Bendroflumethiazide: 1/50 (2) * Cefotaxime: 1/50 (2) * Cetirizine: 1/50 (2) * Ciprofloxacin: 1/50 (2) * Galantamine: 1/50 (2) *	Moderate

Abbreviations: ARIC, Atherosclerosis Risk in Communities; CKD, chronic kidney disease; CPRD, Clinical Practice Research Datalink; DoD, Department of Defence; DoVA, Department of Veterans Affairs; eCrCl, estimated creatinine clearance; ICES, Institute for Clinical Evaluative Sciences; JBI, Joanna Briggs Institute checklist; MDS, Minimum Data Set; NR, not reported; NSAIDs, nonsteroidal anti-inflammatory drugs; OTC, over-the-counter; SR, systematic review; SCREAM, Stockholm CREAtinine Measurements; STOPP, Screening Tool of Older Persons’ Prescriptions; VAs, Veteran Affairs. * Numerator represents the number of patients prescribed each drug inappropriately according to renal function. Denominator represents the total number of individuals who had inappropriate drug dosing according to their renal function. ^¶^ Renal function was assessed using eCrCl (CG equation) and eGFR (MDRD equation). When renal function was determined by the measurement of patient’s eGFR, authors found no cases with inappropriate drug dosing for memantine (*n* = 0) [22].

## Data Availability

All data generated or analysed during this study are included in this published article.

## Supplementary Materials

The following supporting information can be downloaded at https://www.mdpi.com/article/10.3390/jcm13195658/s1, Table S1: Database-specific search strategies; Table S2: Database-specific search syntax; Table S3: Summary of the quality assessment for cross-sectional studies included in our review (*n* = 8); Table S4: Articles that were excluded and the reason for exclusion; Table S5: Complete list of medications examined, including dosage recommendations for their appropriate prescribing according to renal function, for each identified drug from the included studies; and Table S6: The PRISMA 2020 checklist.

## Abbreviations

CKD, chronic kidney disease; CKD-EPI, Chronic Kidney Disease Epidemiology Collaboration; CrCl, creatinine clearance; GFR, glomerular filtration rate; MDRD, Modification of Diet in Renal Disease; NSAIDs, nonsteroidal anti-inflammatory drugs.
