# Supplementary material for: Prevalence and Risk Factors of Inappropriate Drug Dosing among Older Adults with Dementia or Cognitive Impairment and Renal Impairment: A Systematic Review
Source: J Clin Med. 2024 Sep 24;13(19):5658. doi: 10.3390/jcm13195658 (PMC11477088; doi:10.3390/jcm13195658)
Supplement: Supplementary file 1 [file jcm-13-05658-s001.zip › Supplementary Table S1 and Supplementary Table S2 Search strategies.pdf]

Supplementary Table S1. Database-specific search strategies

Research Question	**"What is the prevalence of inappropriate drug dosing and associated factors among older adults with renal impairment and dementia"**			
Concepts	**Inappropriate**	**Drug dosing**	**Renal**	**Dementia**
Synonyms	Appropriate<br>Inappropriate<br>Improper<br>Incorrect<br>Unsuitable<br>Disproportionate<br>Wrong<br>High<br>Decrease<br>Reduce<br>Lower<br>Optimization<br>Adjustment<br>Optimize<br>Adjust<br>Change<br>Risk<br>Preventable<br>Avoidable<br>Unnecessary	Dosage<br>Dose<br>Prescribe/Prescribing<br>Prescription<br>Order<br>Request<br>Chart<br>Administer<br>Use	Kidney impairment<br>Renal<br>impairment<br>Renal insufficiency<br>Kidney insufficiency<br>Kidney disease<br>Renal disease<br>Renal function<br>Kidney function<br>Renal function<br>Kidney function<br>Glomerular filtration rate<br>GFR	Cognitive impairment<br>Alzheimer disease<br>Aphasia<br>Lewy body disease
Search-terms and syntax<br>(e.g. MEDLINE)	inappropriat* or<br>improper* or<br>incorrect* or	dosing* or<br>dosage* or<br>dose* or	renal impair* or<br>kidney impair* or	dementia* or<br>cognitive impair* or<br>alzheimer* or

	unsuitabl* or disproportionat* or wrong* or<br>high* or<br>decreas* or<br>reduc* or<br>lower* or<br>optimiz* or<br>adjust* or<br>chang* or<br>risk* or<br>prevent* or<br>avoid* or<br>unnecessar*	prescri* or<br>order* or<br>request* or<br>chart* or<br>administer* or<br>use*	renal insufficien* or kidney insufficien* or renal disease* or<br>kidney disease* or<br>renal problem* or<br>kidney problem* or<br>renal function* or<br>kidney function* or glomerular filtration rate* or<br>GFR*	aphas* or<br>lewy body*
**Vocabulary terms (database specific)**				
Embase<br>(Emtree)			Renal impairment/ or kidney impairment/ or renal insufficiency/ or kidney insufficiency/ or renal disease/ or kidney disease/ or renal problem/ or kidney problem/ or renal function/ or kidney function/ or glomerular function rate/ or GFR	
Medline<br>(MeSH terms)			Renal impairment/ or Kidney impairment/ or Renal insufficiency/ or Kidney insufficiency/ or Renal disease/ or Kidney disease/ or Renal problem/ or Kidney problem/ or	

			Renal function/ or Kidney function/ or Glomerular function rate/ or GFR	
CINHAL<br><br>(word in subject heading)			Renal impairment OR Kidney impairment OR Renal insufficiency OR Kidney insufficiency OR Renal disease OR Kidney disease OR Renal problem OR Kidney problem OR<br><br>Renal function OR Kidney function OR Glomerular function rate OR GFR	

Supplementary Table S2. Database-specific search syntax

**EMBASE**
Embase <1974 to 2024 August 31><br>1 (inappropriat* or improper* or incorrect* or unsuitabl* or disproportionat* or wrong* or high* or decreas* or reduc* or lower* or optimiz* or adjust* or chang* or risk* or prevent* or avoid* or unnecessar*).mp. [mp=title, abstract, heading word, drug trade name, original title, device manufacturer, drug manufacturer, device trade name, keyword heading word, floating subheading word, candidate term word] 24625153<br>2 limit 1 to (english language and yr="2000 -Current") 19446533<br>3 (dosing* or dosage* or dose* or prescri* or order* or request* or chart* or administer* or use*).mp. [mp=title, abstract, heading word, drug trade name, original title, device manufacturer, drug manufacturer, device trade name, keyword heading word, floating subheading word, candidate term word] 15802614<br>4 limit 3 to (english language and yr="2000 -Current") 12479839<br>5 (renal impair* or kidney impair* or renal insufficien* or kidney insufficien* or renal disease* or kidney disease* or renal problem* or kidney problem* or renal function* or kidney function* or glomerular filtration rate* or GFR*).mp. [mp=title, abstract, heading word, drug trade name, original title, device manufacturer, drug manufacturer, device trade name, keyword heading word, floating subheading word, candidate term word] 701702<br>6 limit 5 to (english language and yr="2000 -Current") 574330<br>7 (dementia* or cognitive impair* or alzheimer* or aphas* or lewy body*).mp. [mp=title, abstract, heading word, drug trade name, original title, device manufacturer, drug manufacturer, device trade name, keyword heading word, floating subheading word, candidate term word] 615200<br>8 limit 7 to (english language and yr="2000 -Current") 524274<br>9 2 and 4 and 6 and 8 4180
**MEDLINE**

Ovid MEDLINE(R) ALL <1946 to August 31, 2024>

1 (inappropriat* or improper* or incorrect* or unsuitabl* or disproportionat* or wrong* or high* or decreas* or reduc* or lower* or optimiz* or adjust* or chang* or risk* or prevent* or avoid* or unnecessar*).mp. [mp=title, book title, abstract, original title, name of substance word, subject heading word, floating sub-heading word, keyword heading word, organism supplementary concept word, protocol supplementary concept word, rare disease supplementary concept word, unique identifier, synonyms, population supplementary concept word, anatomy supplementary concept word] 19102756

2 limit 1 to (english language and yr="2000 -Current") 14489329

3 (dosing* or dosage* or dose* or prescri* or order* or request* or chart* or administer* or use*).mp. [mp=title, book title, abstract, original title, name of substance word, subject heading word, floating sub-heading word, keyword heading word, organism supplementary concept word, protocol supplementary concept word, rare disease supplementary concept word, unique identifier, synonyms, population supplementary concept word, anatomy supplementary concept word] 13890478

4 limit 3 to (english language and yr="2000 -Current") 10123306

5 (renal impair* or kidney impair* or renal insufficien* or kidney insufficien* or renal disease* or kidney disease* or renal problem* or kidney problem* or renal function* or kidney function* or glomerular filtration rate* or GFR*).mp. [mp=title, book title, abstract, original title, name of substance word, subject heading word, floating sub-heading word, keyword heading word, organism supplementary concept word, protocol supplementary concept word, rare disease supplementary concept word, unique identifier, synonyms, population supplementary concept word, anatomy supplementary concept word] 436761

6 limit 5 to (english language and yr="2000 -Current") 291176

7 (dementia* or cognitive impair* or alzheimer* or aphas* or lewy body*).mp. [mp=title, book title, abstract, original title, name of substance word, subject heading word, floating sub-heading word, keyword heading word, organism supplementary concept word, protocol supplementary concept word, rare disease supplementary concept word, unique identifier, synonyms, population supplementary concept word, anatomy supplementary concept word] 401983

8 limit 7 to (english language and yr="2000 -Current") 328228

9 2 and 4 and 6 and 8 1342

CINHAL

S4 ((MH "Dementia") OR "dementia" OR (MH "Frontotemporal Dementia") OR (MH "Dementia, Vascular") OR (MH "Dementia, Multi-Infarct") OR (MH "Lewy Body Disease") OR (MH "Dementia, Senile") OR (MH "Dementia Patients") OR (MH "Mild Cognitive Impairment") OR (MH "Alzheimer's Disease") OR (MH "Aphasia")) AND (S1 AND S2 AND S3) (6)

S3 (MH "Dementia") OR "dementia" OR (MH "Frontotemporal Dementia") OR (MH "Dementia, Vascular") OR (MH "Dementia, Multi-Infarct") OR (MH "Lewy Body Disease") OR (MH "Dementia, Senile") OR (MH "Dementia Patients") OR (MH "Mild Cognitive Impairment") OR (MH "Alzheimer's Disease") OR (MH "Aphasia") (107,788)

S2 (MH "Renal Insufficiency") OR (MH "Kidney Failure, Chronic") OR (MH "Renal Insufficiency, Chronic") OR (MH "Continuous Renal Replacement Therapy") OR "renal impairment" OR (MH "Kidney Diseases") OR "renal disease" OR "renal problem" OR (MH "Kidney Function Tests") OR "renal function" OR (MH "Glomerular Filtration Rate") OR "glomerular filtration rate" OR "GFR" (82,787)<br>S1 "inappropriate dosing" OR (MH "Drug Tapering") OR (MH "Inappropriate Prescribing") (4,365)
**PubMed**
2 Search: (((inappropriat* or improper* or incorrect* or unsuitabl* or disproportionat* or wrong* or high* or decreas* or reduc* or lower* or optimiz* or adjust* or chang* or risk* or prevent* or avoid* or unnecessar*) AND (dosing* or dosage* or dose* or prescri* or deprescri* or order* or request* or chart* or administer* or use*dosing* or dosage* or dose* or prescri* or deprescri* or order* or request* or chart* or administer* or use*)) AND (renal impair* or kidney impair* or renal insufficien* or kidney insufficien* or renal disease* or kidney disease* or renal problem* or kidney problem* or renal function* or kidney function* or glomerular filtration rate* or GFR*)) AND (dementia* or cognitive impair* or alzheimer* or aphas* or lewy body* or creutzfeldt jakob* or progressive supranuclear palsy* or corticobasal degeneration* or multisystem atrophy* or huntington disease*) Filters: English, from 2000 – 2024 (1,873)<br><br>1 Search: (((inappropriat* or improper* or incorrect* or unsuitabl* or disproportionat* or wrong* or high* or decreas* or reduc* or lower* or optimiz* or adjust* or chang* or risk* or prevent* or avoid* or unnecessar*) AND (dosing* or dosage* or dose* or prescri* or deprescri* or order* or request* or chart* or administer* or use*dosing* or dosage* or dose* or prescri* or deprescri* or order* or request* or chart* or administer* or use*)) AND (renal impair* or kidney impair* or renal insufficien* or kidney insufficien* or renal disease* or kidney disease* or renal problem* or kidney problem* or renal function* or kidney function* or glomerular filtration rate* or GFR*)) AND (dementia* or cognitive impair* or alzheimer* or aphas* or lewy body* or creutzfeldt jakob* or progressive supranuclear palsy* or corticobasal degeneration* or multisystem atrophy* or huntington disease*) (2,045)
