# Supplementary material for: Prevalence and Risk Factors of Inappropriate Drug Dosing among Older Adults with Dementia or Cognitive Impairment and Renal Impairment: A Systematic Review
Source: J Clin Med. 2024 Sep 24;13(19):5658. doi: 10.3390/jcm13195658 (PMC11477088; doi:10.3390/jcm13195658)
Supplement: Supplementary file 1 [file jcm-13-05658-s001.zip › Supplementary Table S3 Quality assessment.pdf]

Supplementary Table S3. Summary of the quality assessment for cross-sectional studies included in our review (n = 8)

**Critical appraisal checklist for cross-sectional studies**	**Author (year)**							
	**Delgado et al. 2021**	**Dolder et al. 2009**	**Hanlon et al. 2011**	**Muanda et al. 2019**	**Pfister et al. 2017**	**Schmidt-Mende et al. 2019**	**Secora et al. 2018**	**Sönnerstam et al. 2016**
Were the criteria for inclusion in the sample clearly defined?	Yes	Yes	Yes	Yes	Yes	Yes	Yes	Yes
Were the study subjects and the setting described in detail?	Yes	Yes	Yes	Yes	Yes	Yes	Yes	Yes
Was the exposure measured in a valid and reliable way?	Yes	Unclear	NA	Unclear	NA	Yes	NA	NA
Were objective, standard criteria used for measurement of the condition?	Yes	No	Yes	Yes	Yes	Yes	Yes	Yes
Were confounding factors identified?	NA	NA	NA	NA	NA	NA	NA	NA
Were strategies to deal with confounding factors stated?	NA	NA	NA	NA	NA	NA	NA	NA
Were the outcomes measured in a valid and reliable way?	Yes	Unclear	Yes	Yes	Yes	Yes	Yes	No
Was appropriate statistical analysis used?	Yes	Unclear	Yes	Yes	Yes	Yes	Yes	Yes
**Study quality**	**High**	**Low**	**Moderate**	**Moderate**	**Moderate**	**High**	**Moderate**	**Moderate**

Scores can range from 0 to 8, each question is given a single score if the answer is Yes

Study quality: High quality: score ≥6, Moderate quality: score 4-5, Low quality: score ≤3
