# Supplementary material for: Prevalence and Risk Factors of Inappropriate Drug Dosing among Older Adults with Dementia or Cognitive Impairment and Renal Impairment: A Systematic Review
Source: J Clin Med. 2024 Sep 24;13(19):5658. doi: 10.3390/jcm13195658 (PMC11477088; doi:10.3390/jcm13195658)
Supplement: Supplementary file 1 [file jcm-13-05658-s001.zip › Supplementary Table S4 Articles that were.pdf]

Supplementary Table S4. Articles that were excluded and the reason for exclusion

Article excluded	Reason for exclusion
Memantine-induced myoclonus and delirium exacerbated by trimethoprim (Moellentin et al. 2008)	Case report or <5 cases
Memantine-Induced Myoclonus Precipitated by Renal Impairment and Drug Interactions (Pei et al. 2015)	Case report or <5 cases
A case of amiodarone-induced myoclonus responsive to levetiracetam (Deik et al. 2012)	Case report or <5 cases
Neurologic adverse effects of ranolazine in an elderly patient with renal impairment (Southard et al. 2013)	Case report or <5 cases
Improved renal and cognitive function in a hospice patient after polypharmacy reduction (Whalen et al. 2014)	Case report or <5 cases
Progressive tremor, truncal ataxia, and acute mental status changes after use of buproprion in an 84 year-old man with renal impairment (Rozolsky et al. 2010)	Case report or <5 cases
Hemodialysis for cefepime intoxication: A case report (Mani et al. 2013)	Case report or <5 cases
Can gabapentin be a safe alternative to hormonal therapy in the treatment of inappropriate sexual behavior in demented patients? (Alkhalil et al. 2003)	Case report or <5 cases
Recurrent SSRI-induced myoclonus in a frail octogenarian (Lee et al. 2011)	Case report or <5 cases
The risk of polypharmacy and potentially inappropriate drugs in residential care dementia patients: tips from the PharE study (Gareri et al. 2021)	Inappropriate dosing not referable to renal function
The effect of hospitalization on potentially inappropriate medication use in older adults with chronic kidney disease (Tesfaye et al. 2019)	Inappropriate dosing not referable to renal function
Prevalence of drug-related problems using STOPP/START and medication reviews in elderly patients with dementia (Abramsson et al. 2020)	Inappropriate dosing not referable to renal function
Use of inappropriate medications and their prognostic significance among in-hospital and nursing home patients with and without dementia in Finland (Raivio et al. 2006)	Inappropriate dosing not referable to renal function
Potentially inappropriate medication use and related hospital admissions in aged care residents: the impact of dementia (Eshetie et al. 2020)	Inappropriate dosing not referable to renal function
Drug-related hospital admissions among old people with dementia (Gustafsson et al. 2016)	Inappropriate dosing not referable to renal function
Medication misuse and overuse in community-dwelling persons with dementia (Deardorff et al. 2023)	Inappropriate dosing not referable to renal function
Prescription of Sulphonylureas among Patients with Type 2 Diabetes Mellitus in Italy: Results from the Retrospective, Observational Multicentre Cross-Sectional SUSCIPE (Sulphonyl_UreaS_Correct_Internal_Prescription_Evaluation) Study (Giorda et al. 2020)	Not specific to dementia patients
Rates of Potentially Inappropriate Dosing of Direct-Acting Oral Anticoagulants and Associations With Geriatric Conditions Among Older Patients With Atrial Fibrillation: The SAGE-AF Study (Sanghai et al. 2020)	Not specific to dementia patients
Kidney function and clinical recommendations of drug dose adjustment in geriatric patients (Karsch-Völk et al. 2013)	Not specific to dementia patients
Renal insufficiency and medication in nursing home residents: a cross-sectional study (IMREN) (Hoffmann et al. 2016)	Not specific to dementia patients
Prevalence of potentially inappropriate medications and association with comorbidities in older adults with diabetes in an outpatient visitation setting (Lu et al. 2022)	Not specific to dementia patients
Impact of the Pharmacist Medication Review Services on Drug- Related Problems and Potentially Inappropriate Prescribing of Renally Cleared Medications in Residents of Aged Care Facilities (Gheewala et al. 2014)	Not specific to dementia patients

Intermittent hemodialysis treatment in cefepime-induced neurotoxicity: Case report, pharmacokinetic modeling, and review of the literature (Mani et al. 2015)	Not specific to dementia patients
Capgras delusion with violent behavior in alzheimer dementia: Case analysis with literature review (Kaufman et al. 2014)	Abstract only
Palliative care for an older HIV patient with end-stage renal disease (Ferrari et al. 2014)	Abstract only
A case of subacute encephalopathy, ataxia and myoclonus due to amantadine toxicity in chronic renal insufficiency (Hardwick et al. 2010)	Abstract only
Trimethoprim-sulfamethoxazole should be used with caution in the old-old (Tran et al. 2010)	Abstract only
Donepezil-induced torsades de pointes without QT prolongation (Hadano et al. 2013)	Not specific to renal impairment or diseases
Excessive polypharmacy and potentially inappropriate prescribing in 147 care homes: a cross-sectional study (MacRae et al. 2021)	Not specific to renal impairment or diseases
Nursing-home residents and their drug use: a comparison between mentally intact and mentally impaired residents: The Bergen district nursing home (BEDNURS) study (Nygaard et al. 2003)	Not specific to renal impairment or diseases
Antipsychotic drug use in nursing home residents with and without dementia: keep an eye on the pro re nata medication (Allers et al. 2017)	Not specific to renal impairment or diseases
Ertapenem induced visual hallucinations (Michael et al. 2014)	Wrong study design
New acetylcholinesterase inhibitor (donepezil) treatment for Alzheimer's disease in a chronic dialysis patient (Suwata et al. 2002)	Wrong study design
Cognitive impairment, perceived medication adherence, and high-risk medication use in patients with reduced kidney function: A cross-sectional analysis (Sheets et al. 2022)	No specific drugs with inappropriate dosing listed
Older people with type 2 diabetes, including those with chronic kidney disease or dementia, are commonly overtreated with sulfonylurea or insulin therapies (Hambling et al. 2017)	No data on prevalence of inappropriate drugs prescribing
