# Supplementary material for: Prevalence and Risk Factors of Inappropriate Drug Dosing among Older Adults with Dementia or Cognitive Impairment and Renal Impairment: A Systematic Review
Source: J Clin Med. 2024 Sep 24;13(19):5658. doi: 10.3390/jcm13195658 (PMC11477088; doi:10.3390/jcm13195658)
Supplement: Supplementary file 1 [file jcm-13-05658-s001.zip › Supplementary Table S5 Complete list.pdf]

Supplementary Table S5. Complete list of medications examined, including dosage recommendations for their appropriate prescribing according to renal function, for each identified drug from the included studies

Data recommend avoiding alendronate in people with eCrCl <30 mL/min, but use may be considered in those with eCrCl of >25 to <35 mL/min and without underlying CKD-mineral and bone disorder when the benefits outweigh the risks (44).
Data recommend avoiding alfuzosin in people with eGFR <30 mL/min due to lack of clinical safety in this group of patients (45).
Data shown allopurinol increases risk of allopurinol hypersensitivity syndrome and dose or frequency should adjusted in people with eGFR <50 mL/min (46).
Data recommend to adjust penicillin dose or frequency in people with eGFR <15 mL/min due to risk of crystalluria with high doses (47).
Data recommend to adjust penicillin dose or frequency in people with eGFR <15 mL/min due to risk of crystalluria with high doses (47).
Data recommend close monitoring for baclofen in people with eGFR <30 mL/min due to risk of baclofen-induced neurotoxicity (48).
Data shown diuretic effect of thiazides is lowered in people with eGFR <30 mL/min (49).
Data shown cefotaxime can be administered at a dose of 1 g every 12 hours in people with eCrCl >5 mL/min. In cases of more severe renal failure, the dose should be halved and given every 12 hours (50).
Data recommend to adjust cetirizine dose or frequency in people with eCrCl ≤31 mL/min due to risk of central nervous system depression, sedated state, drowsiness and fatigue (51).
Data recommend close monitoring for ciprofloxacin in people with eGFR <15 mL/min due to risk of ciprofloxacin-induced neurotoxicity (52).
Data recommend avoiding colchicine in people with eCrCl <30 mL/min due to risk of colchicine-induced myotoxicity (53).
Data shown dabigatran increases risk of bleeding in people with eCrCl in the range of 15 to 30 mL/min and use should be avoided in those with eCrCl <15 mL/min (54).
Data shown digoxin increases risk of acute kidney injury in people with eGFR <50 mL/min and dose or frequency should be adjusted based on plasma concentration (47).
Data shown enalapril increases risk of kidney failure in people with eCrCl <10 mL/min (55).
Data shown fondaparinux increases risk of bleeding in people with eCrCl in the range of 30 to <50 mL/min and use of fondaparinux should be avoided in those with eCrCl <30 mL/min (56, 57).
Data shown galantamine use is not recommended in people with eCrCl < 9 mL/min due to risk of central nervous system depression, drowsiness and fatigue (58).
Data recommend avoiding glibenclamide in people with eGFR <60 mL/min due to risk of prolonged hypoglycemia (59).
Data recommend avoiding glipizide in people with eGFR <10 mL/min due to risk of prolonged hypoglycemia (60).
Data shown diuretic effect of thiazides is lowered in people with eGFR <30 mL/min (49).
Data shown NSAIDs increase risk of acute kidney injury in people with eGFR <30 mL/min and prolonged use should be avoided in those with eGFR <60 mL/min (47).
Data shown NSAIDs increase risk of acute kidney injury in people with eGFR <30 mL/min and prolonged use should be avoided in those with eGFR <60 mL/min (47).
Data shown memantine increases risk of neuropsychiatric effects (confusion, dizziness, drowsiness and headache) in people with eGFR <30 mL/min or greater decrements in eGFR (27, 37).
Data shown metformin may increase risk of lactic acidosis in people with eGFR <30 mL/min, but risk-benefit can be considered if eGFR is stable (47).

Data recommend to adjust morphine dose or frequency in people with eGFR <60 mL/min due to risk of central nervous system depression, sedation and respiratory depression and to use morphine with caution in those with eGFR <15 mL/min (61).
Data shown antimicrobial efficacy of nitrofurantoin is decreased in people with eCrCl <30 mL/min (62).
Data shown NSAIDs increase risk of acute kidney injury in people with eGFR <30 mL/min and prolonged use should be avoided in those with eGFR <60 mL/min (47).
Data recommend to use raloxifene with caution in people with eCrCl ≤50 mL/min as raloxifene use was associated with kidney-related adverse events (63).
Data recommend avoiding spironolactone in people with eGFR <30 mL/min due to risk of hyperkalemia (64).
Data recommend avoiding sucralfate in people with eCrCl <30 mL/min as prolonged use can lead to aluminum accumulation (65).
Data recommend to adjust tramadol dose or frequency in people with eGFR <60 mL/min as tramadol lowers the seizure threshold and to use tramadol with caution in those with eGFR <15 mL/min (61).

Abbreviations: eCrCl, estimated creatinine clearance; eGFR, estimated glomerular filtration rate; NSAIDs, nonsteroidal anti-inflammatory drugs.
See main manuscript for references.
